# Metal Interactions in the Ni Hyperaccumulating Population of *Noccaea caerulescens* Monte Prinzera

**DOI:** 10.3390/biology12121537

**Published:** 2023-12-18

**Authors:** Elisa Fasani, Anita Zamboni, Daniela Sorio, Antonella Furini, Giovanni DalCorso

**Affiliations:** 1 Department of Biotechnology, University of Verona, 37134 Verona, Italy; elisa.fasani@univr.it (E.F.); anita.zamboni@univr.it (A.Z.); 2 Centro Piattaforme Tecnologiche, University of Verona, 37134 Verona, Italy; daniela.sorio@univr.it

**Keywords:** hyperaccumulator, *Noccaea caerulescens* Monte Prinzera, hypertolerance, metal transporters

## Abstract

**Simple Summary:**

Hyperaccumulation is a particular trait that has evolved in a few plant species, conferring upon them the ability to accumulate large amounts of metal ions in above-ground tissues without showing symptoms of toxicity. *Noccaea caerulescens* is a model system to study metal hyperaccumulation and hypertolerance, since a significant number of populations exist, characterized by a wide variety of metal tolerance and accumulation. In this report, the serpentine population Monte Prinzera of *N. caerulescens* was treated with excess Ni, Zn and Co and subjected to Fe and Zn deficiency. Transcript analysis, correlated with tissue metal quantification, confirmed that this population was Ni, but not Zn, hypertolerant and hyperaccumulating. Moreover, excess Ni does not induce Fe deficiency as in Ni-sensitive species.

**Abstract:**

Hyperaccumulation is a fascinating trait displayed by a few plant species able to accumulate large amounts of metal ions in above-ground tissues without symptoms of toxicity. *Noccaea caerulescens* is a recognized model system to study metal hyperaccumulation and hypertolerance. A *N. caerulescens* population naturally growing on a serpentine soil in the Italian Apennine Mountains, Monte Prinzera, was chosen for the study here reported. Plants were grown hydroponically and treated with different metals, in excess or limiting concentrations. Accumulated metals were quantified in shoots and roots by means of ICP-MS. By real-time PCR analysis, the expression of metal transporters and Fe deficiency-regulated genes was compared in the shoots and roots of treated plants. *N. caerulescens* Monte Prinzera confirmed its ability to hypertolerate and hyperaccumulate Ni but not Zn. Moreover, excess Ni does not induce Fe deficiency as in Ni-sensitive species and instead competes with Fe translocation rather than its uptake.

## 1. Introduction

To grow and reproduce successfully, living organisms must acquire mineral nutrients that include both metallic and non-metallic elements. Metallic elements, in particular, play a role in plant physiology and biochemistry, participating as components of structural proteins and macromolecules, as regulators of the electrochemical balance between the different cellular compartments and as cofactors of functional enzymes and transcription factors. Therefore, they are involved in all redox reactions, physiological and biochemical cellular processes and organism–environment interactions [1].

In animal and human biology, essential metallic elements include manganese (Mn), iron (Fe), cobalt (Co), copper (Cu), zinc (Zn) and molybdenum (Mo). Metals need to be acquired through the diet; abnormal metal intakes are a common denominator of many illnesses, such as neurodegeneration, cancer, cardiovascular diseases and diabetes [2]. The minerals from rocks, soils and waters are directly absorbed primarily by plants. Regarding plant nutrition, a distinction between macro- and micronutrients is widely accepted, according to the amount of elements required to sustain life and reproduction. Macroelements are needed in relatively large amounts (>1000 mg kg^−1^ dry weight), while Fe, nickel (Ni), Cu, Mn, Zn, Co and Mo are needed in smaller amounts (<100 mg kg^−1^ dry weight) and are thus defined as micronutrients or trace elements (for a detailed review, refer to [3]).

Being sessile organisms, plants have evolved a tightly controlled system to acquire (mainly by root absorption) nutrient elements. It is well known that mineral nutrition is dependent on a highly accurate relationship between the plant roots and the surrounding rhizosphere, i.e., the soil and microorganisms associated with the root [4]. On the other hand, soils can be very diverse in terms of chemical composition. In particular, both natural processes and anthropogenic activities can give rise to the elevated accumulation of essential trace elements in the ground and/or enrich the soils with other minerals, such as cadmium (Cd), mercury (Hg), lead (Pb), chromium (Cr) and arsenic (As), that do not have a biological role and are toxic to plants even at low concentrations. An excess of nutrient metals and toxic elements is detrimental to plants, interfering with cell membrane permeability and integrity, inducing oxidative stress, reacting with sulfhydryl groups and competing with and displacing essential (cat)ionic cofactors in enzymes and signaling components [5]. Despite this evidence, some plant species can live and reproduce on metalliferous soils and enact mechanisms to limit excess metal toxicity. This tolerance can be achieved by two contrasting behaviors. On the one hand, plants can exclude toxic metals from their above-ground tissues by limiting their absorption and/or the root-to-shoot translocation of the metals. These plants are called *excluders*, in view of their capacity to avoid excess metal reaching the most sensitive photosynthetic tissues [6]. Other plant species, falling under the name of metal *hyperaccumulators* [7], can accumulate very large amounts of metal ions in their above-ground tissues, without showing signs of toxicity [8]. The threshold values defining a plant as a hyperaccumulator have been established for each different metal, metalloid and semimetal and are utilized by researchers to identify the plant species that fall into this category (for a review, refer to [9]). As mentioned before, the concentrations of both micronutrients and non-essential metals must be tightly regulated by homeostasis mechanisms. Therefore, most hyperaccumulation mechanisms have likely evolved directly from the latter [10]. The debate regarding the evolutive reasons for the emergence of hyperaccumulation, a trait that displays very high natural selection and rapid evolution [11], was started in 1992 by Boyd and Martens [12], who postulated a variety of theories possibly explaining the evolutive advantages of hyperaccumulation. Among these, the most widely accepted is the *elemental defense hypothesis*; according to the latter, hyperaccumulation is described as an effective mechanism protecting plants against herbivore and pathogen attacks [13]. Similarly, an allelopathic advantage has been suggested as a positive result of hyperaccumulation. Indeed, when metal-enriched dead plant tissues are cycled back into the soil, the release of toxic elements and their accumulation in the soil top layer might negatively affect competitors’ germination or survival [14].

Plant metal hyperaccumulation has widely attracted the attention of researchers; indeed, its direct implication as a source of information and genetic material for biotechnological approaches makes it valuable in view of the phytoremediation of metal-polluted lands and waters or of the phytomining of valuable metals from soils [15].

The Brassicaceae family is particularly rich in hyperaccumulating and hypertolerant species, including the model species *Arabidopsis halleri*, as well as many members of the *Noccaea* and *Alyssum* genera [9]. The facultative metallophyte *Noccaea caerulescens*, formerly known as *Thlaspi caerulescens*, is probably one of the best known and studied examples of hyperaccumulators. It has evolved mainly in three different types of habitat in Europe: (*i*) non-metalliferous soils; (*ii*) calamine soils, enriched in Zn, Pd and Cd; and (*iii*) serpentine soils, rich in Ni. Members of the three edaphic groups are phenotypically different and show diverse metal accumulation and tolerance properties, mainly towards Zn, Cd and Ni [16,17]. Very interestingly, in this species, Zn hyperaccumulation is considered to be spread species-wide and characterized by variations in degree between local populations, while the Cd, Pb, and Ni hyperaccumulation capacities seem to be population-specific, both in the native environment and under laboratory conditions [17]. For instance, *N. caerulescens* growing on calamine soil near Ganges (France, defined hereafter as GA population) is a well-known Cd hyperaccumulator. The population growing in calamine soil next to Trooz-Prayon (Belgium), and therefore known as Prayon, is characterized by relatively low Cd accumulation and a very poor Ni accumulation capacity [17]. The French population Puy de Wolf (PdW, France) grows on ultramafic soils and is able to competitively accumulate Zn and Ni [18]. The population that is the subject of the current work—MP—is a Ni hyperaccumulating ecotype growing on the ultramafic soils of Monte Prinzera in the Italian Apennines (44.65096°lon–10.08369°lat), a site that is poorly vegetated and characterized by the presence of serpentine rock outcroppings, with low organic matter content and high Ni and Fe levels [19].

The genetic determinants underlying the ability of metal accumulation have been, for a long time, a matter of research. It appears that metal hyperaccumulators have independently evolved common distinctive traits, such as enhanced metal absorption by the roots, followed by efficient translocation to the shoot, where metal ions are compartmentalized and detoxified, ensuring elevated metal tolerance [20].

Recent research activity has focused on the molecular biology of hyperaccumulation and hypertolerance. For instance, it has been suggested that Ni and Zn could share the same low-affinity transport system for root internalization, a hypothesis also supported by isotope fractionation analysis [16]. Interestingly, the overexpression of metal transporters, due to a combination of enhanced promoter activity and gene duplications, was demonstrated to confer hyperaccumulation ability. For example, Milner et al. [21] showed that in the roots and shoots of the calamine *N. caerulescens* GA, the expression of the metal transporter *NRAMP1* was five-fold higher than in the Prayon population, largely lacking the ability to accumulate Cd. The copy number for *NRAMP1* in the GA population was also approximately four times higher than in Prayon [21]. A similar phenomenon was proposed for HMA3, a vacuolar transporter involved in Cd sequestration and tolerance [22]. Nevertheless, most research has investigated the mechanisms for Zn and Cd accumulation in calamine metallophytes; on the other hand, little is known about the exact determinants of Ni tolerance and accumulation at the molecular level, despite Ni metallophytes constituting the great majority of metal hyperaccumulators [9,23].

It has been shown that besides metal availability in the growth substrate, inter-element interactions have a great effect on actual accumulation in shoots. For example, Zn and Fe inhibit Ni uptake in the Zn/Ni hyperaccumulator *Thlaspi pindicum* [24]. The interaction between different nutrients and/or trace elements implies that different elements possibly share common pathways for uptake, translocation and homeostasis, resulting in competition for the same transporters [18,25]. Interestingly, RNA-seq analysis, comparing the root transcriptome in three *N. caerulescens* populations with contrasting metal accumulation capacity, suggested that transporters IRT1, ZIP10 and vacuolar IREG2 might be associated with Ni accumulation in the MP population [26]. In the present work, we analyzed metal accumulation and gene expression in MP plants grown in a hydroponic culture for three weeks. Unlike the similarly serpentine population PdW, which showed a strong interaction between Fe and Ni and the responses of Fe deficiency upon Ni treatment [18], *N. caerulescens* MP does not sense Ni exposure as Fe deprivation. Indeed, in MP plants kept under high Ni concentrations, the expression of *IRT1* was not modulated in comparison to control conditions. On the other hand, *IREG2* expression in roots was highly reduced upon Ni exposure in comparison with the shoot expression level, pointing to the higher availability of Ni for root-to-shoot translocation. Moreover, conversely to PdW, MP does not show a preference towards Zn accumulation. Overall, this evidence indicates the high plasticity of related plant taxa to adapt independently to highly selective environments, enacting different, and sometimes contrasting, strategies to cope with similarly challenging edaphic conditions.

## 2. Materials and Methods

### 2.1. Plant Material and Growth Conditions

Plants were grown from seeds of *Noccaea caerulescens* subsp. *caerulescens* ecotype Monte Prinzera (*N. caerulescens* MP), derived from a natural population identified in the MP4 site in the Monte Prinzera Natural Reserve (Parma Province, Italy [19]). After quick sterilization, seeds were laid on standard MS medium and stratified at 4 °C for four weeks [27]. After stratification, plates were moved to a growth chamber at 22 °C/18 °C day/night temperatures with a 16-h photoperiod. Three-week-old seedlings were transferred to 1-liter polyethylene pots filled with modified half-strength Hoagland’s nutrient solution, as described in [28]. Ni was also added to the hydroponic solution at a concentration of 10 μM, which has been proven to be beneficial for the growth of *N. caerulescens* MP [26]. After one week of acclimation to standard hydroponic growth conditions, the growth substrate was changed, applying the following treatments:

(1) **Control (Ctrl)**: half-strength nutrient solution amended with 10 μM NiSO_4_;
(2) **100 μM Ni**: half-strength nutrient solution amended with 100 μM NiSO_4_;
(3) **−Zn**: half-strength nutrient solution lacking Zn salts and amended with 10 μM NiSO_4_;
(4) **−Fe**: half-strength nutrient solution lacking Fe salts and amended with 10 μM NiSO_4_;
(5) **100 μM Zn**: half-strength nutrient solution amended with 10 μM NiSO_4_ and 100 μM ZnSO_4_;
(6) **50 μM Co**: half-strength nutrient solution amended with 10 μM NiSO_4_ and 50 μM CoSO_4_.

**Figure 1:**
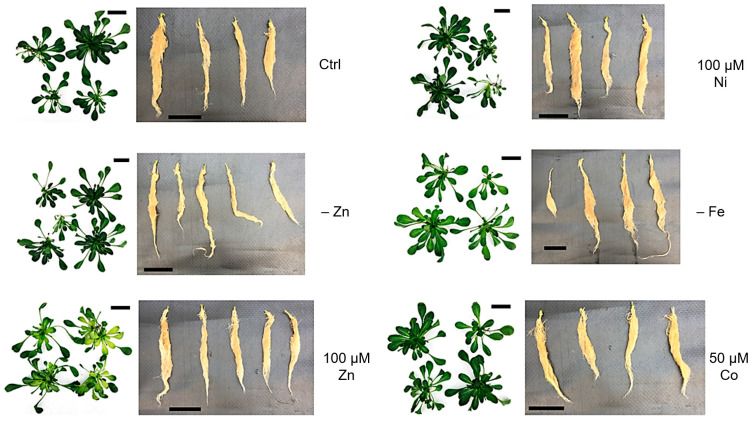
Phenotypes of *N. caerulescens* MP plants after growth for three weeks in hydroponic solution, modified as reported on the right side of each panel. Each panel shows rosettes (**left picture**) and roots (**right picture**) from one pot, representative of three biological replicates. Black bars in the panels correspond to 5 cm.

Plants were grown for a further three weeks in the growth chamber. Air bubbling was applied in each pot to ensure root oxygenation, and the solution was regularly changed every five days. Four to five plants were allocated to each pot, and each treatment pot was produced in triplicate. The plant phenotype after three weeks of growth is shown in Figure 1 (one pot for each condition is reported as a representative). At the end of the experiment, plants were harvested, and shoots and roots were separated. Shoots were briefly washed in bi-distilled cold water and wiped dry, frozen in liquid nitrogen and stored at −80 °C until use. Roots were washed for 5 minutes in ice-cold 50 mM Na–EDTA to remove eventual ions adsorbed to the root surface (as described in [29]) and rinsed in ice-cold bi-distilled water, wiped dry, immediately frozen in liquid nitrogen and stored at −80 °C until use.

### 2.2. RNA Extraction and Gene Expression Analysis

Total RNA was extracted from frozen leaf and root tissue using the TRIzol reagent (Thermo Fisher Scientific, Waltham, MA, USA). After DNase treatment, first-strand cDNA was synthesized using the enzyme SuperScript II Reverse Transcriptase (Thermo Fisher Scientific) according to the product protocol. Real-time RT-PCR (40 amplification cycles) was carried out with the Applied Biosystems QuantStudio 3 (Applied Biosystems, Foster City, CA, USA) using Platinum SYBR Green qPCR SuperMix UDG (Thermo Fisher Scientific). Each reaction was performed in triplicate and melting curves were analyzed to ensure the amplification of a single product. Each of the three pots for each treatment was treated as a biological replicate. Quantitative data were normalized to the endogenous reference genes α-tubulin (*TUB*) and elongation factor 1α (*EF1α*) of *N. caerulescens* (genome accession GenBank GCA_900406465.1, released July 2019). The 2^−ΔΔCT^ method was used for the analysis of relative gene expression levels [30]. Genes investigated were FeR-like regulator of iron uptake *FIT1*, the iron regulated transporter *IREG2*, the heavy metal ATPase 4 (*HMA4*), two basic helix-loop-helix *bHLH38* and *bHLH39*, the Zn transporter *ZIP10*, the Fe transporter *IRT1*, the sulfate transporter *SULTR1,1* and the citrate root xylem loading transporter *FRD3*. Specific primers used are reported in Table 1; primer efficiency was determined using the LinRegPCR v. 2012.2 program [31].

### 2.3. Metal Content Quantification and Calculation of the Translocation Factors

Frozen tissues were oven-dried at 60 °C. The content of Ni, Zn, Cu, Fe, Mn and Co was determined by means of inductively coupled plasma mass spectrometry (ICP-MS). Briefly, dried shoot and root tissues (about 10 mg) were mineralized in a 3-mL TFM sampling insert (Milestone, Sorisole, Italy), adding 250 µL of HNO_3_ (69% ultrapure grade, Romil, Cambridge, UK). The reactions were carried out at 180 °C for 20 min using a microwave digestor. Three inserts were placed in a TFM 100-mL vessel with 11 mL of Milli-Q water and 1 mL of ultrapure-grade hydrogen peroxide (30%, Romil, Cambridge, UK). Samples were diluted to 2% HNO_3_ with ultrapure-grade water (18.2 MΩ·cm at 25 °C). Finally, multielemental analysis of samples was performed by means of the Agilent 7500ce ICP-MS detection system (Agilent Technologies, Santa Clara, USA). Calibration curves were obtained by diluting a custom-made multielement standard (Romil). Measurement accuracy and matrix effect errors were checked using a standard reference material (NIST 1515 apple leaves, Merck KGaA, Darmstadt, Germany). The translocation factor for each metal was calculated as the ratio between the metal concentration in the leaf tissue and the concentration in the root [32].

### 2.4. Chlorophyll Quantification

Frozen leaves were ground to power in liquid nitrogen, aliquoted and weighted. Pigments were extracted with 80% aqueous acetone containing 2.5 mM sodium phosphate buffer—pH 7.8. Concentrations for chlorophyll *a* and chlorophyll *b* were determined spectrophotometrically by measuring absorbance at 646.6 and 663.6 nm (blank at 750 nm) and adopting the classical equations described in [33].

### 2.5. Quantification of Metal Chelating Agents

First, 50 mg of frozen shoot and root tissue was ground in liquid nitrogen. Then, 1.5 mL of HPLC-grade water was added to each sample. After brief vortexing, samples were sonicated in an ultrasonic bath in ice-cold water for 10 min and incubated for 15 min on ice. Afterwards, samples were centrifuged at 14,000× *g* rpm at 4 °C for 10 min. Supernatants were filtered through 0.2-µm filters. The samples were diluted with HPLC-grade water and analyzed by UltiMate 3000 (Thermo Fisher Scientific, Milan, Italy) coupled to the triple quadrupole mass spectrometer TSQ Fortis (Thermo Fisher Scientific). Because of the different chemical structures, two analytical methods were developed: one for the identification of nicotianamine (NA) and the other for malic and citric acids. For both methods, chromatographic separation was carried out with the column Luna^®^ 3 μm 100 Å, LC Column 150 × 4.6 mm (Phenomenex Ltd., Aschaffenburg, Germany). The mobile phase for both analyses consisted of phase A: 0.1% (*v*/*v*) formic acid in deionized water and phase B: methanol. For the analysis of NA, 5 μL of sample was injected into the column using a flow rate of 750 μL min^−1^. The gradient was programmed as follows: from 1% to 95% B within 7 min, plus 3 min of equilibration. Electrospray ionization was set in positive ion mode, using the following source parameters: 355 °C for the ion transfer tube temperature, 350 °C for the vaporizer temperature, 60 for the sheath gas/arb pressure, 15 for the auxiliary gas/arb pressure and 3500 V for the spray voltage. For the analysis of malic and citric acids, 5 μL of sample was injected into the column using a flow rate of 500 μL min^−1^. The gradient was programmed as follows: from 50% to 95% B within 5 min, plus 3 min of equilibration. Electrospray ionization was set in negative ion mode, using the following source parameters: 300 °C for the ion transfer tube temperature, 350 °C for the vaporizer temperature, 50 for the sheath gas/arb pressure, 10 for the auxiliary gas/arb pressure and 3500 V for the spray voltage. The analyses were performed in multiple reaction monitoring (MRM) mode using the following ion transitions: NA, 304.1 → 114.1, 185.1, 286.2; malic acid, 133.1 → 71.1, 115; citric acid, 190.9 → 84.8, 87, 111.1. Data acquisition and handling were performed with the Chromeleon Chromatography Studio software 7 (Thermo Fisher).

### 2.6. Statistical Analysis

Data in histograms are represented as the mean ± standard deviation in elemental analysis and as the mean ± standard error in real-time RT-PCR. The statistical significance of experimental data and Pearson correlation on metal content were calculated using GraphPad Prism 7 (GraphPad Software, Boston, MA, USA). All analyses were evaluated by means of a one-way ANOVA followed by a post hoc Tukey’s test. Statistically significant variations at *p* < 0.05 are marked with letters, with the same letter corresponding to non-statistically significant differences.

## 3. Results

### 3.1. Phenotypic Analysis of Plants

After three weeks of treatment as described in Section 2, plants were harvested, and shoots and roots separated and photographed. The addition of 100 μM Ni to the growth medium did not produce any visible phenotype either in roots or in shoots (Figure 1 and Figure S1). On the other hand, the most visible phenotypes were due to Fe deficiency and excess Zn, with the latter causing severe leaf chlorosis, as confirmed by measuring the chlorophyll content (Figure S1B). While excess Zn induced chlorosis in younger leaves, probably as a sign of toxicity, Fe deficiency-induced more evident chlorosis in older leaves (Figure 1 and Figure S1A). Zn deficiency and excess Co did not produce any visible phenotype in treated plants after three weeks of culture, except for a moderate reduction in chlorophyll *a* in both and the light rolling and wilting of the leaf margins upon Co treatment (Figure S1B).

Interestingly, no effect was induced in the roots, except for the slightly enhanced growth of the roots of plants kept in Fe deficiency.

### 3.2. Metal Concentration in Shoots of N. caerulescens MP

After three weeks of treatment, plants were harvested and shoot and root tissues were separated and briefly washed to remove residual nutrient solution, as described in the Materials and Methods, and the Ni, Cu, Zn, Fe, Mn and Co concentrations were measured by means of ICP-MS.

**Figure 2:**
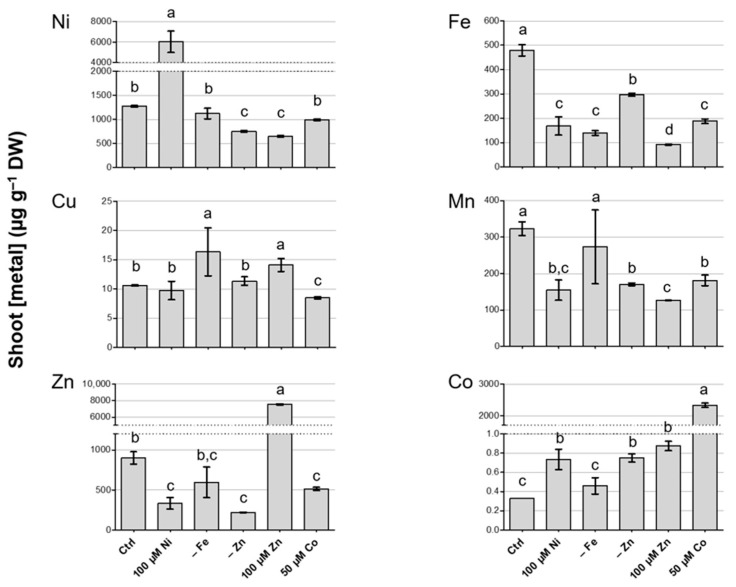
Metal quantification in shoots of *N. caerulescens* MP after three weeks in hydroponic solution modified as reported in Materials and Methods; treatments are listed on the *x*-axis. The measured metal is indicated on the graph. The represented values are means ± SD. Different letters above the histograms indicate statistical significance, evaluated by one-way ANOVA followed by a post hoc Tukey’s test (*p* < 0.05).

In shoots, the different treatments had a significant effect on the metal content when compared to control conditions (Figure 2). First, it is worth noting that the Ni content was higher than 1000 µg g^−1^ DW in most of the growth conditions tested. Although the hyperaccumulation thresholds refer to plants grown in their native habitats, the values achieved in this work, also when not under Ni excess, fit well with those characterizing strong Ni hyperaccumulators [34]. When Ni was supplied in the highest concentration (100 µM), *N. caerulescens* MP accumulated up to 6 mg g^−1^ Ni DW. In the same conditions, the accumulation of Co was enhanced, whereas Fe, Mn and Zn were accumulated to a lower extent.

Zn deficiency also influenced the overall metal accumulation in the shoots of *N. caerulescens* MP. As expected, when compared to the control solution (containing 2 µM Zn [28]), the accumulation of Zn was drastically reduced, and this was responsible for the modest leaf chlorosis in the above-ground tissues (Figure S1). Secondly, the accumulation of Mn, Fe and Ni was negatively influenced by Zn deficiency, whereas Co accumulated to a higher level than in control conditions. Fe deficiency had a limited influence on metal accumulation, apart from Fe itself, which was reduced, and Cu, which was in present a greater amount in Fe-deficient shoots. Interestingly, no effect on Ni accumulation was observed upon Fe deficiency.

Zn excess, as with Ni excess, induced a reduction in Fe accumulation; Mn and Ni were also accumulated at lower levels in the shoots of plants treated with 100 µM Zn. Notably, under this condition, the amount of Zn accumulated was only slightly higher than the amount of Ni accumulated in Ni excess (ca. 7 mg g^−1^ DW), but it was combined with an evident phenotype of toxicity. Furthermore, Zn exceeded the hyperaccumulation threshold in shoots [34] only upon Zn excess.

Finally, excess Co also produced Co accumulation exceeding hyperaccumulation levels [34], without significant toxicity symptoms. Furthermore, this condition influenced Fe, Mn and Zn, reducing their accumulation, while it had no effect on Ni accumulation. Cu accumulation was mostly uninfluenced by the different metal treatments, and, when modulated, the difference from control conditions was modest.

### 3.3. Metal Concentration in Roots of N. caerulescens MP and Effect on Root-to-Shoot Translocation

**Figure 3:**
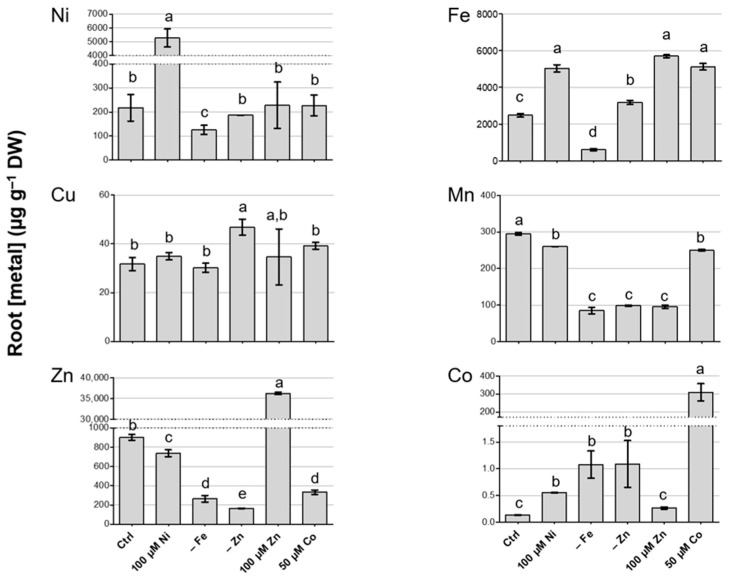
Metal quantification in roots of *N. caerulescens* MP after three weeks in hydroponic solution modified as reported in Materials and Methods; treatments are listed on the *x*-axis. The measured metal is indicated on the graph. The represented values are means ± SD. Different letters above the histograms indicate statistical significance, evaluated by one-way ANOVA followed by a post hoc Tukey’s test (*p* < 0.05).

Metal quantification was also performed in root tissues (Figure 3). Similarly to shoots, Cu accumulation in the roots was modulated only by Zn deficiency, while the other treatments did not have significant effects. Excess Ni determined a decrease in Zn and Mn, as in the shoot. Interestingly, Fe accumulation was enhanced by 100 µM Ni, whereas, in shoots, it was lower in the same conditions; Ni and Co were also detected in greater amounts. Excess Zn had a positive effect on Fe accumulation and a negative one on Mn. Both Zn and Fe deficiency induced a significant reduction in Mn and Zn root adsorption and enhanced Co acquisition. Conversely, Fe deficiency negatively impacted Ni accumulation, whereas Zn deficiency did not produce any significant effect on it. Moreover, Fe acquisition was moderately enhanced by Zn deficiency, while low Fe in the growth substrate corresponded to low Fe in the root tissues. Finally, Co excess affected Zn and Mn accumulation, which were reduced, and Fe accumulation, which was enhanced (Figure 3).

The effect of the different treatments on metal root-to-shoot translocation was evaluated by calculating the translocation factors (TFs, Table 2). As previously highlighted with reference to Ni and Zn accumulation upon Ni and Zn excess, respectively, *N. caerulescens* MP greatly prefers Ni over Zn. Consistently, the Ni TFs exceeded 1 under all treatments considered, whereas the Zn TFs were higher than 1 only upon Zn and Fe deficiency and Co excess, and the Zn TF upon Zn excess was very low (0.2). Indeed, upon 100 µM Ni, the Ni concentration in the roots was lower than in the shoots, while, upon 100 µM Zn, the Zn in the roots was almost five times higher than the Zn concentration in the shoots.

Moreover, the Co TFs were above 1 in control conditions and in all excess treatments, and they reached a maximum in Co excess, where, indeed, consistent accumulation in shoots was achieved.

Pearson correlation was applied to identify direct interactions between metals in uptake and translocation. In the analysis, the metal content in the roots and shoots was plotted against the element concentrations in the growth media (Supplementary Tables S1 and S2) and against each other (Supplementary Table S3). In both roots and shoots, Ni, Zn and Co linearly correlated with the corresponding concentrations in the medium, as expected (Tables S1 and S2); analogously, the shoot concentrations of these metals correlated positively with the concentrations in the roots (Table S3). On the other hand, the Fe content correlated significantly only in the root vs. medium comparison (Table S1), whereas, in shoots, the correlation coefficient was not significant (Tables S2 and S3). When different metals were considered, no interaction was identified in roots. Interestingly, Cu negatively correlated with Fe in leaves (Table S2). In the shoot vs. root comparison, a moderate correlation was found between Mn in the roots and Cu in the shoots, and between Fe in the roots and Mn in the shoots, although the results were not statistically significant (Table S3).

### 3.4. Gene Expression Analysis

To better understand the genetic determinants underlying the specific metal accumulation in *N. caerulescens* MP, we analyzed the steady state expression of genes that encode for proteins and transporters involved in metal homeostasis. Transcript levels for transporters ZIP10, IRT1 and IREG2 and for transcription factors FIT1, bHLH38 and bHLH39 were analyzed in the roots and shoots of *N. caerulescens* MP plants after the three-week application of the growth conditions tested. Due to its importance in Zn and Fe translocation, as involved in the xylem loading of metal–citrate complexes [35], the expression of the citrate efflux transporter FRD3 was also monitored. ZIP10, a putative Zn transporter [36]; IRT1, involved in Fe uptake in roots [37]; and IREG2, a vacuolar metal transporter involved in Ni detoxification [38], were proposed as associated with Ni hypertolerance/accumulation in *N. caerulescens* [26]. Both *IRT1* and *IREG2* are regulated by FIT1 (FER-like Fe deficiency-induced transcription factor) upon Fe deficiency [38]. FIT1 performs its regulatory activity in complex with, among others, bHLH38 and bHLH39, also induced by Fe deficiency [39,40,41].

**Figure 4:**
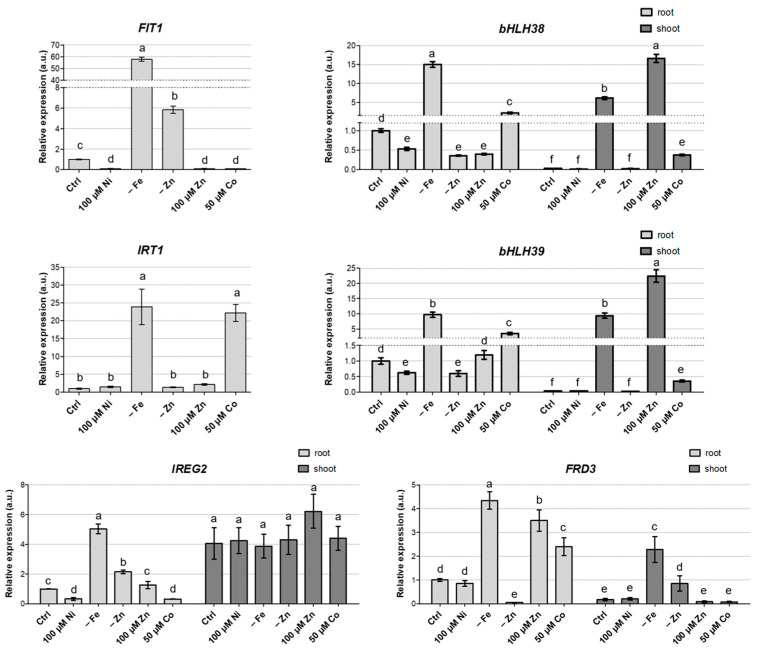
Real-time RT-PCR analysis of the expression of *FIT1* and *IRT1* in roots and *bHLH38*, *bHLH39*, *IREG2* and *FRD3* in the shoots and roots of *N. caerulescens* MP after three weeks of growth in hydroponic solution modified as indicated in the *x*-axis labels. Values refer to the expression levels of each gene in the roots of plants treated with 10 µM Ni, set as 1. Different letters above the histograms indicate statistical significance, evaluated by one-way ANOVA followed by a post hoc Tukey’s test (*p* < 0.05).

In the shoots, *ZIP10*, *IRT1* and *FIT1* transcripts were undetected in any of the conditions tested. In the roots, on the contrary, the expression of *FIT1*, *bHLH38* and *-39*, *IRT1, IREG2* and *FRD3* was greatly enhanced upon Fe deficiency (Figure 4).

Interestingly, both *FIT1* and *IREG2* were upregulated also in Zn deficiency, but the same condition did not influence *IRT1* or *bHLH38*–*39* expression. Noticeably, excess Ni did not induce the expression of any of the Fe-responding genes considered in the roots, and, in fact, *FIT1* and *IREG2* expression was even reduced in this condition. Of particular interest is the case of excess Co. In the roots, this condition induced the expression of *bHLH38* and *bHLH39* (Figure 4), albeit to a lesser extent if compared to Fe deficiency, but this was not coupled with an increase in *FIT1* mRNA abundance. *IRT1* and *FRD3* were also significantly upregulated in the roots of Co-treated plants; this evidence could explain the increased Fe accumulation in roots under these growth conditions (Figure 3).

Regarding the Zn transporter *ZIP10*, its expression in roots was only moderately modulated by the different growth substrates, except for excess Co and Zn (Figure S2); in these conditions, the doubled expression of *ZIP10* was observed. However, such expression modulation does not fully correlate with the Zn accumulation in shoots or roots in the different growth conditions.

In the shoot, *IREG2* expression was constant, regardless of the growth substrate (Figure 4), while, in the roots, it was influenced by the metal content, and it showed a great reduction upon Ni and Co excess. On the other hand, the shoot accumulation of *bHLH38*, *bHLH39* and *FRD3* transcripts was strongly upregulated upon Fe deficiency; a significant positive modulation was also observed for the two transcription factors upon Zn excess (Figure 4).

Notably, both Zn and Co excess resulted in the enhanced expression of the sulfate transporter *SULTR1.1* in roots (Figure S2). Together with SULTR1.2, the protein has been characterized as a high-affinity sulfate transporter, localized in the cortex, epidermis and root hairs and usually detectable upon S deficiency [42] and reportedly under the excess of some metall(oid)s, such as Zn, Cd, Cr and Se [43,44,45,46]. Finally, the transcripts of metal transporter HMA4, involved in the xylem loading of metals, were also detected at high levels in roots and modulated by the different treatments (Figure S2) in a way that was partially consistent with the corresponding Zn TFs (Table 2). In particular, *HMA4* was downregulated in Zn excess, where the Zn TF was the lowest, and upregulated upon Fe deficiency and Co excess, corresponding to the increased Zn TFs (Figure S2 and Table 2).

### 3.5. Accumulation of Chelating Compounds

Most metal ions are not free in plant tissues and cells but are bound to ligands that protect the plant metabolisms from the toxic effects of free metal ions. Organic acids, such as malate (Mal) and citrate (Cit), nicotianamine (NA) and histidine, are the main chelating compounds that have been proposed to have a role in the vacuole storage or root-to-shoot transport of metals [47].

**Figure 5:**
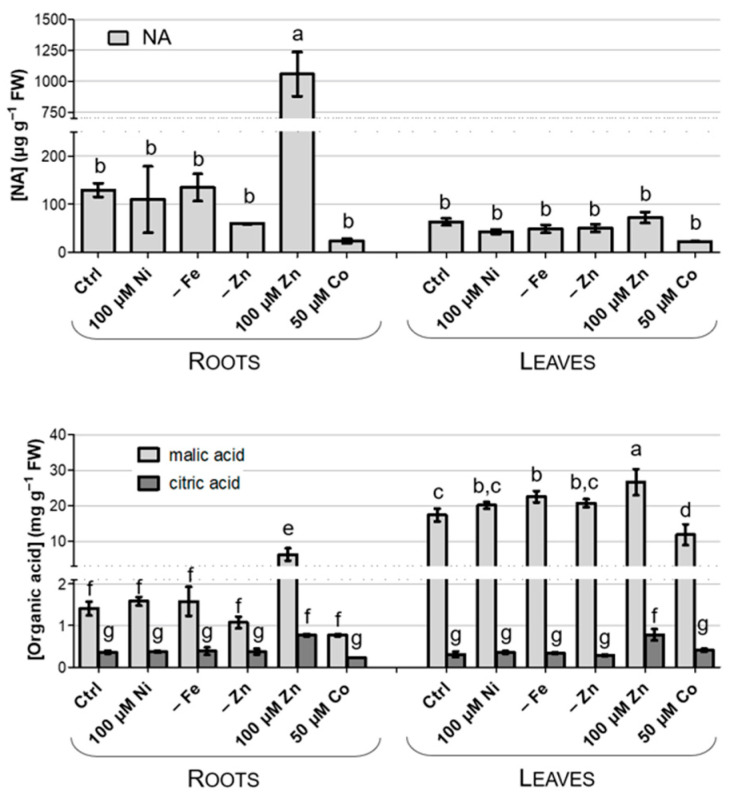
Quantification of nicotianamine (NA), citric acid and malic acid in roots and leaves of *N. caerulescens* MP treated as indicated. Different letters above the histograms indicate statistical significance, evaluated by one-way ANOVA followed by a post hoc Tukey’s test (*p* < 0.05).

NA, Cit and Mal were quantified in the shoots and roots of *N. caerulescens* MP. As reported in Figure 5, only Zn excess seems to enhance NA, Mal and Cit root abundance. The NA abundance in leaves is lower if compared with the root values, pointing to a role for this chelating compound in the root-to-shoot translocation of metals, rather than in their storage in the vacuoles, as already suggested by other authors [47]. Interestingly, Mal is relatively more abundant than Cit, and it accumulates preferentially in leaves, while Cit, the less abundant ligand, accumulates at comparable amounts in leaves and roots (Figure 5).

## 4. Discussion

Element homeostasis in plants is an extremely complex network, due both to the need to keep the level of nutrients and non-essential elements under tight control and to the interaction of different elements with others [48]. In *N. caerulescens*, independent adaptation to diverse metal-rich environments has led to the evolution of ecotype-specific mechanisms for ion homeostasis [17,26,49]. Among the various populations, the one evolved on the serpentine Monte Prinzera is of particular interest for its significant ability to hypertolerate and hyperaccumulate Ni, both in native conditions and in laboratory experiments [19,50].

In this work, *N. caerulescens* MP confirmed its ability to hypertolerate and hyperaccumulate Ni but not Zn, as indicated by its general phenotype, metal shoot concentration and translocation factors. Despite exceeding the Zn hyperaccumulation threshold [36] upon Zn excess, in this experiment, MP showed toxicity symptoms upon Zn excess and only moderate Zn accumulation in all other treatments, as well as low TFs; in fact, this phenotype is inconsistent with the hyperaccumulation trait. This evidence deviates from what was observed in the similarly serpentine population from Puy de Wolf (France), which shows a preference towards Zn accumulation [18]. Differences in their behavior towards metals were also found in global investigations of several *N. caerulescens* accessions that have evolved in different edaphic environments. In these contexts, the MP population showed significantly higher Zn and Ni accumulation than PdW when grown in spiked soil [16], and comparable Zn accumulation and significantly higher Ni accumulation when cultivated in hydroponics [17], suggesting that the two populations may have evolved different mechanisms to cope with metal excess, despite the common ultramafic background. Indeed, a genetic analysis of *N. caerulescens* populations in France proposed that the adaptation to specific metalliferous environments occurred after the divergence of geographically distinct genetic subunits [49]. Together with the Ni-associated traits, the current experiments showed that MP has an enhanced ability to tolerate and accumulate excess Co. Co accumulation has yet been poorly investigated; however, ultramafic soils are generally rich in Co, and indeed the Co concentration in the rocks and rhizosphere of Monte Prinzera is about 100–200 ppm [51]. The high Co accumulation of MP upon Co excess is compatible with the results achieved in PdW, which showed both similar tolerance and accumulation [18]. In particular, the Co shoot concentration in MP exceeded the hyperaccumulation threshold [23] upon Co excess, and its TFs were above 1 in most treatments, supporting the strong ability of *N. caerulescens* MP to transfer Co to the shoots. The combined hyperaccumulation of Ni and Co was discovered in other serpentine metallophytes, such as *Berkheya coddii* [52], *Glochidion* cf. *sericeum* [53], *Alyssum murale* and *Alyssum corsicum* [54,55]. Contrary to *N. caerulescens* PdW [18], Co accumulation resulted in decreased Zn uptake and Fe translocation, but did not impact Ni accumulation in either roots or shoots. Although Ni and Co were proposed to compete for the same transporters in some serpentine species [52,54,56], the results here presented support the fact that Ni and Co follow independent routes for Ni and Co transport and storage in this population [55].

The mechanisms for Ni uptake and translocation are still poorly understood, although research has given moderate insights regarding the process [57,58]. The overlapping of Ni management with the homeostatic networks of other metals has been proposed; in some plant species, interactions were found between Ni and Fe, Cu and/or Mn [59,60,61,62]. In *N. caerulescens*, Ni was described as interacting with Mn and Zn at the species level [16,49], and with Fe and Mn in *N. caerulescens* PdW [18]. In MP and in the conditions tested here, no significant correlation was found between Ni and other nutrients in either the roots or shoots. However, Ni excess strongly reduced the Fe, Zn and Mn accumulation in the shoots, and Fe deficiency decreased the Ni levels in the same tissue. Overall, despite the lack of significance in the correlation, the results support the existence of some form of interference between Ni and other micronutrients also in this population; however, the absence of a clear trend does not allow us to determine whether such interaction is synergistic or competitive.

Among the possible interference points, chelating agents are one of the most likely. Characterized by constitutively elevated concentrations in hyperaccumulators [63], Cit, Mal and other chelating compounds (NA and His) are known to bind metal ions in the plant tissues or cells, to protect the plant metabolisms from the toxic effects of free metal ions and to play a role in vacuole metal storage or root-to-shoot transport. Although great species specificity has been observed, in *N. caerulescens*, Ni has been proposed to preferentially bind to histidine, an event that prevents root retention and allows translocation towards the shoot [7], while NA is thought to be involved in Zn tolerance and accumulation [26], although a role in Ni binding has been proposed in the calamine population of *N. caerulescens* Les Malines [64]. Furthermore, organic acids have been shown to be a factor in Ni accumulation: Mal was proposed as the main Ni storage chelator in serpentine *N. caerulescens, Alyssum murale* and *Leptoplax emarginata* [63], while Ni seems to be mainly associated with citrate in the shoots of *Alyssum* and *T. goesingense* [7] and in the stems of *A. murale* and *L. emarginata* [63]. In this context, genes involved in Mal metabolism and transport were proposed as determinants for Ni tolerance in *N. caerulescens* MP [26]. In our conditions, no modulation of Mal accumulation was detected upon Ni excess, whereas the accumulation of this organic acid in shoots was constitutively high, consistent with a role in Ni compartmentalization and tolerance. On the other hand, both Cit and NA were suggested as associated with Zn accumulation and tolerance [26], and indeed both ligands were accumulated at higher levels in excess Zn. Consistently, *FRD3* expression in roots was mainly induced by both Fe deficiency and the treatments with excess Zn and Co, which induced a reduction in Fe content in shoots. FRD3 is a member of the multidrug and toxin efflux (MATE) family and encodes for a Cit transporter that has been reported as implicated in Fe transport via the xylem [65], as well as in Zn translocation and tolerance [66]. Despite the absence of conclusive evidence regarding metal chelators, it should be remembered that their levels, as in the case of Mal, may be constitutively high as a result of the adaptation of the MP population to serpentine environments.

Remarkably, we still do not know the exact nature of Ni uptake transporters. It has been proposed that Ni may enter through Fe transporters, and that Ni-induced Fe deficiency is responsible for the higher Fe and Ni accumulation observed in *N. caerulescens* [29]. Consistently with this hypothesis, the transcript levels for metal transporters IRT1 and IREG2 were suggested to correlate with Ni tolerance in *N. caerulescens* MP [26]. IRT1 is a plasma membrane transporter involved in Fe uptake and is highly induced upon Fe deficiency [67]. In *A. thaliana*, IRT1 was proposed to mediate Ni accumulation thanks to its low substrate specificity and its upregulation upon Ni-induced Fe deficiency [37]. Similarly, IREG2, mediating ion translocation across the tonoplast, has been indicated as an important determinant in Ni tolerance and accumulation [38]. In the Ni-sensitive species *A. thaliana*, *IREG2* is induced, together with *IRT1*, as a response to the Ni-induced Fe deficiency [29,38]. In the Ni-tolerant excluder *Noccaea japonica*, originating from a serpentine soil, *IREG2* expression is elevated in the roots, and this seems to be responsible for the compartmentalization of toxic Ni ions in the vacuoles of root cells, preventing their translocation to the shoots [29]. As supported by the current experiment, the hyperaccumulator *N. caerulescens* MP, also adapted to a serpentine soil, likely evolved a contrasting mechanism of tolerance that allows for Ni translocation to the shoot by maintaining lower *IREG2* expression in the roots and reducing retention in this organ. On the contrary, *IREG2* expression is higher and mostly constant under the different treatments in the shoot, the final destination of Ni translocation, where IREG2 may play a key role in Ni detoxification. Similar results were reported for the *N. caerulescens* Ganges population [29].

Although Ni-dependent Fe deficiency was demonstrated in *N. caerulescens* PdW by the upregulation of *IRT1* [18], in *N. caerulescens* MP, Ni excess does not induce true Fe deficiency. Indeed, the treatment with 100 µM Ni generated a reduction in shoot Fe content but an increase in root Fe content, suggesting that, in the MP population, Ni competes with Fe translocation rather than its uptake. A similar behavior was observed also upon Ni excess in *A. thaliana* [37,68].

Although, both in *A. thaliana* and *N. caerulescens* PdW, *IRT1* is moderately upregulated upon Ni excess [18,37], neither *IRT1* nor *IREG2* expression is induced in roots upon 100 µM Ni in *N. caerulescens* MP. It should be noticed that both *IRT1* and *IREG2* are greatly upregulated in the roots in the absence of Fe, showing that, also in *N. caerulescens* MP, these genes are associated with sensing and the response to Fe deficiency, as in *A. thaliana* [38,67]. Furthermore, also the expression of the transcription factors *FIT1*, *bHLH38* and *bHLH39*, part of the key regulatory hub for Fe deficiency responses and directly modulating *IRT1* and *IREG2* [40,41,69], shows a significant increase upon Fe deprivation. Notably, neither transcription factor was induced upon Ni excess, further supporting that *N. caerulescens* MP does not undergo Ni-dependent Fe deficiency. In fact, *FIT1* and *IREG2* expression was even reduced in this condition, and indeed their expression profiles in the roots were overall similar, suggesting that, also in *N. caerulescens* MP, *IREG2* is regulated by the FIT1 transcription factor, as in *A. thaliana* [38]. However, it should be noted that the transcript levels of all regulatory and target genes were not completely equivalent: for example, *IRT1* expression upon Co excess is not explainable by the regulation of the Fe deficiency hub. Analogously, *IREG2* was constitutively expressed in the shoots, whereas, in this tissue, *FIT1* transcripts are absent and *bHLH38/39* are variably modulated upon different metal treatments. However, it is possible that other currently unknown regulatory nodes intervene in these target genes in tissues (e.g., shoots) and conditions (e.g., Co excess) where the FIT1–bHLH38/39 complex is not active.

Finally, both Zn and Co excess resulted in enhanced expression in the roots of transcript of the sulfate transporter *SULTR1,1*. SULTR1,1 was proposed among the putative determinants for Ni accumulation in *N. caerulescens* MP [26], although no positive modulation was observed upon Ni excess in our experiment. On the other hand, it is possible that its expression correlates rather with the ability to tolerate and accumulate Co in this serpentine population, as supported by its upregulation upon Co excess. Zn excess enhanced the expression of *SULTR1* also in *Macleaya cordata*, a metal-tolerant species found in Chinese mining areas, able to accumulate Zn, Hg, Cd, Pb and Mn [43,44]. Such enhanced sulfur uptake can be stimulated by an increased demand in the biosynthesis of protecting S-containing ligands (e.g., glutathione) involved in Zn and Co detoxification [70].

## 5. Conclusions

In this study, the cross-interaction between Ni management and the homeostasis of metallic nutrients was investigated in the serpentine *N. caerulescens* Monte Prinzera. This population was confirmed as a Ni hyperaccumulator and a strong Co accumulator. Both excesses induced alterations in the levels of Fe and Mn, suggesting the existence of interactions in the homeostasis of the different metals in this population; however, current evidence does not allow us to determine the type of interference. Notably, the MP population does not undergo the gene expression modulation associated with the Ni-dependent Fe deficiency response, as instead happens in non-tolerant *A. thaliana* and serpentine *N. caerulescens* Puy de Wolfe. In conclusion, these results confirm the independent evolution of diverging strategies to cope with Ni excess in *N. caerulescens* populations from different genetic units.

## Figures and Tables

**Table 1:** Genes analyzed in the presented study and specific primers for real-time RT-PCR amplification.

Target Gene	Primer in Forward	Primer in Reverse
*FIT1*	CTCTAACCTAAGCTCTCCTTC	AAGTGATCCAGTGATCCACAG
*IREG2*	CATGCTCATGGCTGGAGTTG	CCAAAAGATTAGCCATCAAGT
*HMA4*	GTGGCAGAAGAGTTACTTCGA	TTTGGAACGGGGAGATGAGG
*bHLH38*	GTCTCTTCAGAGGGAAATGAG	TCTGAGGCTGGAAGGCACG
*bHLH39*	AGGGAAATGGAATAGACAACC	CCGTCTGAGGAATACTTAGCT
*ZIP10*	TCCTCCAGGCAGAATACACG	TGTTATGAGAGAGGAAGGGCT
*IRT1*	CCGACGGGAACATTTTCACC	GAAATTTGTGCCACGGGTTCT
*SULTR1,1*	CAGTTGACAGTCCCGCTGAA	GCTGTTGGAGAGCGATTGTG
*FRD3*	TGTGTTAGGACTTGGACTGTC	AGATGCTCCAAAATTGACTC
*TUB*	CCTACGCACCAGTCATCTCT	CGAGATCACCTCCTGGAACA
*EF1α*	GGATACAAATGAAGAAGAGAGG	CCAGCACACCAATGTCCGC

**Table 2:** Translocation factors (TFs) calculated as ratio between the metal concentration in the leaves and the metal concentration in the root, both expressed in µg/g, ± standard deviations. Different letters within a column indicate significant differences among the treatments (*p* < 0.05). To highlight the effect of the treatment on the TFs, background colors are used to define ranges for each TF, as reported in the legend.

	Ni	Cu	Zn	Fe	Mn	Co
**10 µM Ni**	6.02 ± 1.51 a	0.34 ± 0.03 a	0.10 ± 0.05 a	0.19 ± 0.01 a	1.09 ± 0.05 a	2.53 ± 0.14 a
**100 µM Ni**	1.14 ± 0.06 b	0.28 ± 0.03 a	0.45 ± 0.12 b	0.04 ± 0.01 b	0.59 ± 0.11 a	1.32 ± 0.18 b
**−Zn**	4.01 ± 0.10 c	0.24 ± 0.01 b	1.34 ± 0.05 c	0.09 ± 0.01 c	1.73 ± 0.07 b	0.74 ± 0.26 b
**−Fe**	9.07 ± 2.23 a	0.54 ± 0.10 c	2.23 ± 0.43 d	0.23 ± 0.01 d	3.58 ± 0.75 c	0.43 ± 0.30 c
**100 µM Zn**	3.14 ± 1.40 c	0.44 ± 0.18 a,c	0.21 ± 0.01 e	0.02 ± 0.00 b	1.32 ± 0.06 b	3.34 ± 0.42 a
**50 µM Co**	4.44 ± 0.94 a,c	0.22 ± 0.00 b	1.55 ± 0.05 c	0.04 ± 0.00 b	0.73 ± 0.05 a	7.51 ± 1.63 e

## Data Availability

Data are contained within the article and supplementary materials.

## Supplementary Materials

The following supporting information can be downloaded at: https://www.mdpi.com/article/10.3390/biology12121537/s1, Figure S1: Phenotype and chlorophyll quantification in treated plants; Figure S2: Expression of *ZIP10*, *HMA4* and *SULTR1*; Table S1: Pearson correlation coefficients for metal concentrations in roots, as compared to metal concentration in the growth substrate; Table S2: Pearson correlation coefficients for metal concentrations in shoots, as compared to metal concentration in the growth substrate; Table S3: Pearson correlation coefficients for metal concentrations in shoots and roots.
