# Supplementary material for: Metal Interactions in the Ni Hyperaccumulating Population of Noccaea caerulescens Monte Prinzera
Source: Biology (Basel). 2023 Dec 18;12(12):1537. doi: 10.3390/biology12121537 (PMC10740792; doi:10.3390/biology12121537)
Supplement: Supplementary file 1 [file biology-12-01537-s001.zip › biology-2758298-supplementary.pdf]

Supplementary information

A B

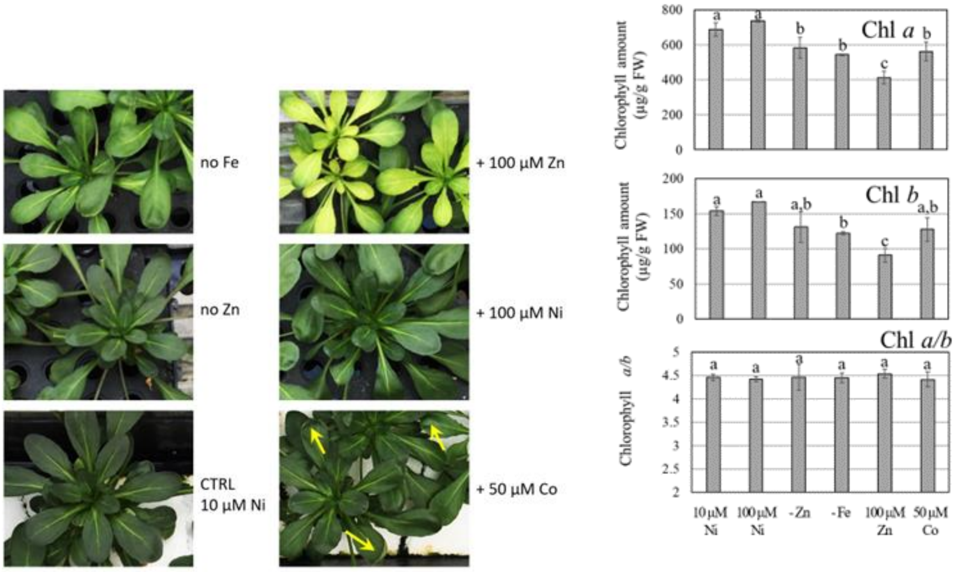

Figure S1. (**A**) Close-up photograph of the phenotype for plants treated as indicated on the right side of each panel. Wilting and rolling of leaf margins due to excess Co is marked by yellow arrows. (**B**) Chlorophyll analysis of grounded aerial tissues of plants treated as indicated. Values reported are the means ± SD of three technical replicates of each biological replicate. Chlorophylls were extracted in buffered aqueous acetone is 80% aqueous acetone containing 2.5 mM sodium phosphate buffer pH 7.8 to minimize conversion of chlorophylls to pheophytins and chl *a* and chl *b* concentrations were spectrophotometrically as described in [21].

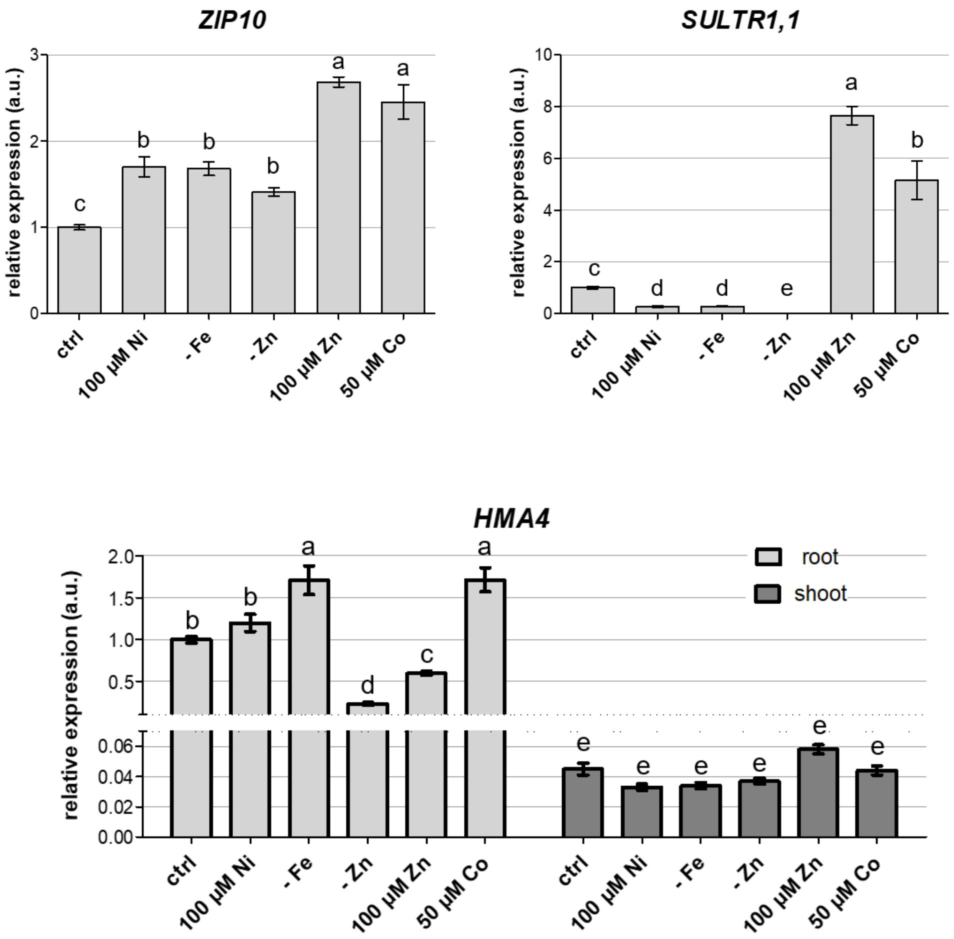

Figure S2. Real Time RT-PCR analysis of *ZIP10* and *SULTR1* expression in roots and *HMA4* in roots and shoots of *N. caerulescens* MP treated as indicated. Values are relative to the expression level of the gene in plants treated with 10 µM Ni, set as 1. Different letters above the histograms indicate statistical significance, evaluated by on-way ANOVA followed by a *post hoc* Tukey's test (p < 0.05).

Table S1. Pearson correlation coefficients for metal concentrations in roots, as compared to metal concentration in the growth substrate. Statistically significant coefficients are in bold and marked with asterisks. P-values (in italics) are reported under the coefficient.

		**Metal in roots**					
		**Mn**	**Fe**	**Ni**	**Cu**	**Zn**	**Co**
**Metal in the growth medium**	**Ni**	0.399	0.337	**0.995 *****	-0.092	-0.191	-0.199
		0.199	*0.284*	*< 0.001*	*0.776*	*0.551*	*0.536*
	**Fe**	0.482	**0.771 ****	0.216	0.412	0.207	0.197
		0.112	*0.003*	*0.501*	*0.183*	*0.518*	*0.540*
	**Zn**	-0.418	0.504	-0.187	-0.121	**1.000 *****	-0.195
		0.177	*0.095*	*0.561*	*0.708*	*< 0.001*	*0.543*
	**Co**	0.348	0.359	-0.192	0.204	-0.205	**0.993 *****
		0.267	*0.252*	*0.550*	*0.524*	*0.523*	*< 0.001*

Table S2. Pearson correlation coefficients for metal concentrations in shoots, as compared to metal concentration in the growth substrate. Statistically significant coefficients are in bold and marked with asterisks. P-values (in italics) are reported under the coefficient.

		Metal in shoots					
		**Mn**	**Fe**	**Ni**	**Cu**	**Zn**	**Co**
**Metal in the growth medium**	**Ni**	-0.275	-0.202	**0.982 *****	-0.305	-0.230	-0.200
		0.387	*0.528*	*< 0.001*	*0.336*	*0.472*	*0.533*
	**Fe**	-0.376	0.303	0.158	**-0.687** *	0.185	0.200
		0.229	*0.338*	*0.624*	*0.014*	*0.565*	*0.533*
	**Zn**	-0.424	-0.472	-0.262	0.350	**0.997 *****	-0.195
		0.170	*0.121*	*0.411*	*0.265*	*< 0.001*	*0.544*
	**Co**	-0.129	-0.136	-0.189	-0.492	-0.199	**1.000 *****
		0.689	*0.674*	*0.557*	*0.105*	*0.535*	*< 0.001*

Table S3. Pearson correlation coefficients for metal concentrations in shoots and roots. Statistically significant coefficients are in bold and marked with asterisks. P-values (in italics) are reported under the coefficient.

		Metal in shoots					
		**Mn**	**Fe**	**Ni**	**Cu**	**Zn**	**Co**
**Metal in shoot**	**Mn**	1					
	**Fe**	0.667	1				
		0.147					
	**Ni**	-0.221	-0.140	1			
		0.673	*0.791*				
	**Cu**	0.166	-0.387	-0.350	1		
		0.754	*0.449*	*0.497*			
	**Zn**	-0.438	-0.430	-0.294	0.399	1	
		0.385	*0.395*	*0.571*	*0.433*		
	**Co**	-0.152	-0.136	-0.191	-0.549	-0.199	1
		-0.152	*0.797*	*0.717*	*0.260*	*0.705*	
		**Metal in roots**					
		**Mn**	**Fe**	**Ni**	**Cu**	**Zn**	**Co**
**Metal in root**	**Mn**	1					
	**Fe**	0.245	1				
		0.640					
	**Ni**	0.409	0.352	1			
		0.421	*0.493*				
	**Cu**	-0.193	0.308	-0.106	1		
		0.714	*0.552*	*0.842*			
	**Zn**	-0.413	0.507	-0.184	-0.139	1	
		0.415	*0.304*	*0.727*	*0.793*		
	**Co**	0.347	0.357	-0.193	0.246	-0.206	1
		0.501	*0.487*	*0.714*	*0.639*	*0.695*	
		**Metal in roots**					
		**Mn**	**Fe**	**Ni**	**Cu**	**Zn**	**Co**
**Metal in shoot**	**Mn**	0.292	-0.799	-0.329	-0.519	-0.497	-0.152
		0.575	*0.056*	*0.524*	*0.292*	*0.315*	*0.774*
	**Fe**	0.521	-0.346	-0.200	0.070	-0.462	-0.137
		0.289	*0.502*	*0.704*	*0.896*	*0.356*	*0.796*
	**Ni**	0.462	0.269	**0.993 *****	-0.174	-0.262	-0.191
		0.357	*0.607*	*< 0.001*	*0.742*	*0.617*	*0.717*
	**Cu**	-0.791	-0.531	-0.352	-0.426	0.384	-0.548
		0.061	*0.278*	*0.494*	*0.399*	*0.453*	*0.261*
	**Zn**	-0.394	0.473	-0.222	-0.187	**0.997 *****	-0.201
		0.440	*0.343*	*0.672*	*0.723*	*< 0.001*	*0.703*
	**Co**	0.348	0.359	-0.193	0.245	-0.205	**1.000 *****
		0.498	*0.484*	*0.714*	*0.640*	*0.697*	*< 0.001*
